# Overexpression of HSPB6 inhibits osteosarcoma progress through the ERK signaling pathway

**DOI:** 10.1007/s10238-023-01216-9

**Published:** 2023-10-20

**Authors:** Liangyu Guo, Kangwen Xiao, Yuanlong Xie, Zhiqiang Yang, Jun Lei, Lin Cai

**Affiliations:** Department of Spine Surgery and Musculoskeletal Tumor, Zhongnan Hospital of Wuhan University, Wuhan, China

**Keywords:** Osteosarcoma, HSPB6, Proliferation, Metastasis, Apoptosis, ERK signaling pathway

## Abstract

Heat shock protein B6 (HSPB6) plays a certain role in the formation of several cancers, whereas its effect on osteosarcoma remains unclear. In this study, the effect of HSPB6 on osteosarcoma was validated through numerous experiments. HSPB6 was down-regulated in osteosarcoma. As indicated by the result of CCK-8 and colony formation assays, HSPB6 overexpression was likely to inhibit the osteosarcoma cells proliferation, whereas the flow cytometry analysis suggested that apoptosis of osteosarcoma cells was increased after HSPB6 overexpression. Furthermore, transwell and wound healing assays suggested that when HSPB6 was overexpressed, osteosarcoma cells migration and invasion were declined. Moreover, the western blotting assay suggested that the protein level of p-ERK1/2 was down-regulated in osteosarcoma when HSPB6 was overexpressed. Besides, the effect of HSPB6 on osteosarcoma in vivo was examined. As indicated by the result, HSPB6 overexpression was likely to prevent osteosarcoma growth and lung metastasis in vivo. As revealed by the findings of this study, HSPB6 overexpression exerted anticancer effects in osteosarcoma through the ERK signaling pathway and HSPB6 may be suitable target for osteosarcoma molecular therapies.

## Introduction

Osteosarcoma has been confirmed as the most prevalent primary malignant bone cancer. It originates from mesenchymal stem cells while predominantly affecting young adults and children [1]. Moreover, osteosarcoma is characterized by fast development, a high metastatic potential, as well as a poor prognosis [2]. Besides, patients suffering from osteosarcoma may develop additional symptoms related to certain pathological characteristics [3]. The metastasis and relapse have been reported as the main cause of death in osteosarcoma patients [1]. The prognosis for osteosarcoma patients can be notably improved through the development of chemotherapeutics and surgical operation [4]. Nevertheless, the drug resistance can result in a poor prognosis of osteosarcoma therapy while reducing the survival rate of osteosarcoma patients [5]. Thus, the mechanism of osteosarcoma development should be investigated, and potential treatment targets for osteosarcoma patients should be identified [6].

Heat shock proteins, conserved molecular chaperone proteins, were initially considered as stress-responsive proteins required for the survival of organisms or cells under thermal stress. Subsequently, existing research has suggested that a variety of injuries such as stimuli, including oxidative stress, inflammation, and ischemia may produce heat shock proteins [7]. In addition, heat shock proteins have a wide variety of roles including freshly translated peptide chain folding, protein trafficking, protein complex synthesis, immunomodulation, and developmental regulation [8]. It has been discovered that heat shock proteins play vital roles in tissue malignancies [9–13]. HSPB6, also termed HSP20, serves as a remarkable member of heat shock protein family and showed scarce expression in human hepatocellular carcinoma (HCC) cell lines. Next, HCC cells development is suppressed by HSPB6 overexpression [14]. Moreover, HCC cells overexpressing HSPB6 can lead to the increase of the higher levels of cleaved caspase-7, cleaved caspase-3, and cleaved PARP. Besides, the PARP cleavage can expedite cellular breakdown, thus serving as a hallmark for apoptotic cells [15]. Moreover, bladder cancer cells transfected with HSPB6 are capable of limiting cell migration [16]. Furthermore, SKOV3 and A2780 ovarian cancer cells are grown with the chemotherapeutic medication cisplatin to develop cisplatin-resistant versions known as SKOV3/CDDP and A2780/CDDP cells. As revealed by existing research, these cisplatin-resistant cells express significantly less HSPB6 protein expression [17].

Mitogen-activated protein kinase (MAPK) cascades can facilitate the cellular processes such as cell differentiation, proliferation, and apoptosis. Besides, three major pathways are involved in MAPK pathway including extracellular signal-related kinase (ERK), the stress-activated protein kinase/Jun-N-terminal kinase (SAPK/JNK) and the p38 MAPK and an interesting target for developing anticancer drugs is ERK [18, 19] Existing research has suggested that ERK is correlated with osteosarcoma progression. Niclosamide repressed the osteosarcoma progression and also suppressed the ERK signaling pathway in osteosarcoma [20]. Moreover, the suppression of miR-765 can lead to the decreased ERK signaling pathway while inhibiting proliferation, migration, and invasion in osteosarcoma cells [21]. Besides, suppression of ERK signaling pathway enhance the anti-osteosarcoma activity of autophagy inhibitors [22].

According to previous publications, HSPB6 showed a correlation with a wide variety of malignancies. However, the association between osteosarcoma and HSPB6 remained unclear. Therefore, we explored the effect of HSPB6 on osteosarcoma. And we found that HSPB6 overexpression decreased osteosarcoma progression through the ERK signaling pathway.

## Materials and methods

### Tissue collection

The osteosarcoma tissues and normal adjacent tissues were collected from patients in Zhongnan Hospital of Wuhan University. Tissues were stored in liquid nitrogen for subsequent experiment. Written informed consent was acquired from all the patients. The study was approved by the Institutional Ethics Committee of Zhongnan Hospital of Wuhan University.

### Cell lines and culture

The human osteoblast cell line hFOB 1.19 and human osteosarcoma cell line MNNG/HOS were purchased from Cell Bank of Chinese Academy of Sciences. hFOB 1.19 cells were cultured in DMEM/F-12 medium whereas MNNG/HOS cells were cultured in MEM medium. The human osteosarcoma cell line U2OS was provide by BeNa Culture Collection and cultured in McCoy’s 5A medium. All media were supplemented with the 10% FBS and 1% penicillin–streptomycin. MNNG/HOS and U2OS cells were maintained in an incubator at 37 °C with 5% CO_2_, whereas hFOB1.19 cells were maintained at 33.5 °C with 5% CO_2_.

### Construction of HSPB6 overexpression in osteosarcoma cells

Hanbio (Shanghai, China) provided the vector plasmid, HSPB6 overexpression plasmid, vector lentivirus and HSPB6 overexpression lentivirus. For transfection, U2OS and MNNG/HOS cells were transfected with plasmids using GP-transfect-Mate. For infection, U2OS and MNNG/HOS cells were infected with lentivirus using polybrene, and infected osteosarcoma cells were selected with the use of puromycin.

### RNA extraction and quantitative real-time PCR (RT-qPCR)

The total RNA of osteosarcoma cells was extracted using the TRIzol reagent (ELK Biotechology, China). Next, cDNA was synthesized from total RNA by using a reverse transcriptase kit (Vazyme, China). The RT-qPCR was performed by SYBR green mixture (Vazyme, China). The 2^−ΔΔCt^ method was employed to examine the fold change and the GAPDH serve as an internal reference gene. Besides, GAPDH and HSPB6 primers are listed in Table 1.

**Table 1:** The primer sequences

Primer name	Sequence
GAPDH-Forward	5′-GGAGCGAGATCCCTCCAAAAT-3′
GAPDH-Reverse	5′-GCTGTTGTCATACTTCTCATGG-3′
HSPB6-Foward	5′-ACGCTCGCCCCCTACTACCT-3′
HSPB6-Reverse	5′-CGACGAATCCGTGCTCATCC-3′

### Western blotting

Using the RIPA Lysis Buffer (Sevicebio, China) supplemented with a protease suppressor cocktail and phosphatase suppressor cocktail (MCE, China), proteins from osteosarcoma cells were isolated and the concentration of proteins were examined following the bicinchoninic acid (BCA) assay. Subsequently, proteins were used SDS-PAGE gels for separation, transferred to polyvinylidene difluoride (PVDF) membranes, and membranes were incubated with primary antibodies against GAPDH, HSPB6, ERK1/2 and p-ERK1/2 overnight at 4 °C after blocked with 5% skimmed milk. After being washed with TBST, membranes were incubated with horseradish peroxidase-linked secondary antibody. Furthermore, proteins were visualized with an ECL Kit (Vazyme, China).

### Cell counting kit-8 (CCK-8) assay

Osteosarcoma cells were digested using trypsin solution and then seeded in 96-well plates with 2 × 10^3^ cells per well. The volume of per well reached 200 μL of complete medium. Next, the CCK-8 reagent was incubated with osteosarcoma cells in an incubator for 2 h. Lastly, the absorption was determined at 450 nm using a microplate reader.

### Colony formation assay

The osteosarcoma cells were evenly planted in 6-well plates at 1 × 10^3^ cells per well. Moreover, medium was changed every three days. Subsequently, osteosarcoma cells were washed in Phosphate-Buffered Saline (PBS), followed fixation with 4% paraformaldehyde and stained with crystal violet. The colonies were photographed and then tallied.

### Flow cytometry

The osteosarcoma cells were inoculated in 6-well plates. After being rinsed with PBS, cells were suspended in 1 × Binding buffer (MultiSciences, China). After Annexin V-PE and 7-AAD were stained (MultiSciences, China), the cell suspension was incubated in the dark circumstance, and then the apoptosis was identified.

### Transwell assay

The chamber (Corning, USA) for the cell migration assay or the chamber precoated with Matrigel (Corning, USA) for the cell invasion assay. Briefly, the 4 × 10^4^ osteosarcoma cells with serum-free medium seeded into upper chamber. And bottom chamber covered complete medium. Subsequently, the migrated or invaded cells were fixed and stained with crystal violet. Lastly, osteosarcoma cells were washed and then imaged using the inverted microscope.

### Wound healing assay

Osteosarcoma cells were cultured in complete medium in 6-well plates. Subsequently, the 200 µL sterile pipette tip was adopted to create gaps. Cells were washed with PBS and cultured in serum-free media. Next, the gaps were observed and imaged at 0, 24, and 48 h using the inverted microscope.

### RNA-sequencing

Total RNA from osteosarcoma cells was extracted and then employed for RNA-sequencing (RNA-seq). The RNA-seq was performed and then analyzed by Genechem (Shanghai, China).

### Xenograft model

The animal experiment gained approval from Experimental Animal Welfare Ethics Committee of Zhongnan Hospital of Wuhan University. In brief, 4-week-old male BALB/c-nude mice were provided by GemPharmatech. For subcutaneous tumor-bearing model, 5 × 10^6^ MNNG/HOS cells were subcutaneously injected into the nude mice. For tail vein injection lung metastasis model, 2 × 10^6^ MNNG/HOS cells were injected into the tail of nude mice. Lastly, nude mice were sacrificed. Tumor and lung tissues were harvested, and tumor were weighed, photographed and followed by immunohistochemistry and TUNEL assays, the lung tissues were collected and followed by hematoxylin and eosin (H&E) staining.

### Immunohistochemistry (IHC)

The tumor and normal tissues were embedded in paraffin, and sections were deparaffinized and rehydrated. Subsequently, sections were subjected to antigenic repair and administrated with 3% H_2_O_2_. After blocked with BSA, the sections were incubated with primary antibodies against HSPB6, PCNA and MMP-2 overnight at 4 °C. Subsequently, sections were administrated with the secondary antibody. Besides, the sections were stained with DAB solution, and nucleus counterstaining was used for hematoxylin.

### TUNEL assay

The apoptosis of the tumors was assessed through TUNEL staining in accordance with the guideline of the manufacturer. In general, sections were stained in dark circumstance with TUNEL solution. Subsequently, the nuclei counterstaining was employed for DAPI.

### Statistical analysis

The statistical analysis was conducted using GraphPad Prism 8.0 software. The data are expressed as means and standard deviations (SD) from three independent experiments. Student's* t*-test was performed to determine statistical significance between the two groups.* P*-value < 0.05 indicated a difference with statistical significance.

## Results

### HSPB6 was down-regulated in several tumors and osteosarcoma

**Fig. 1:**
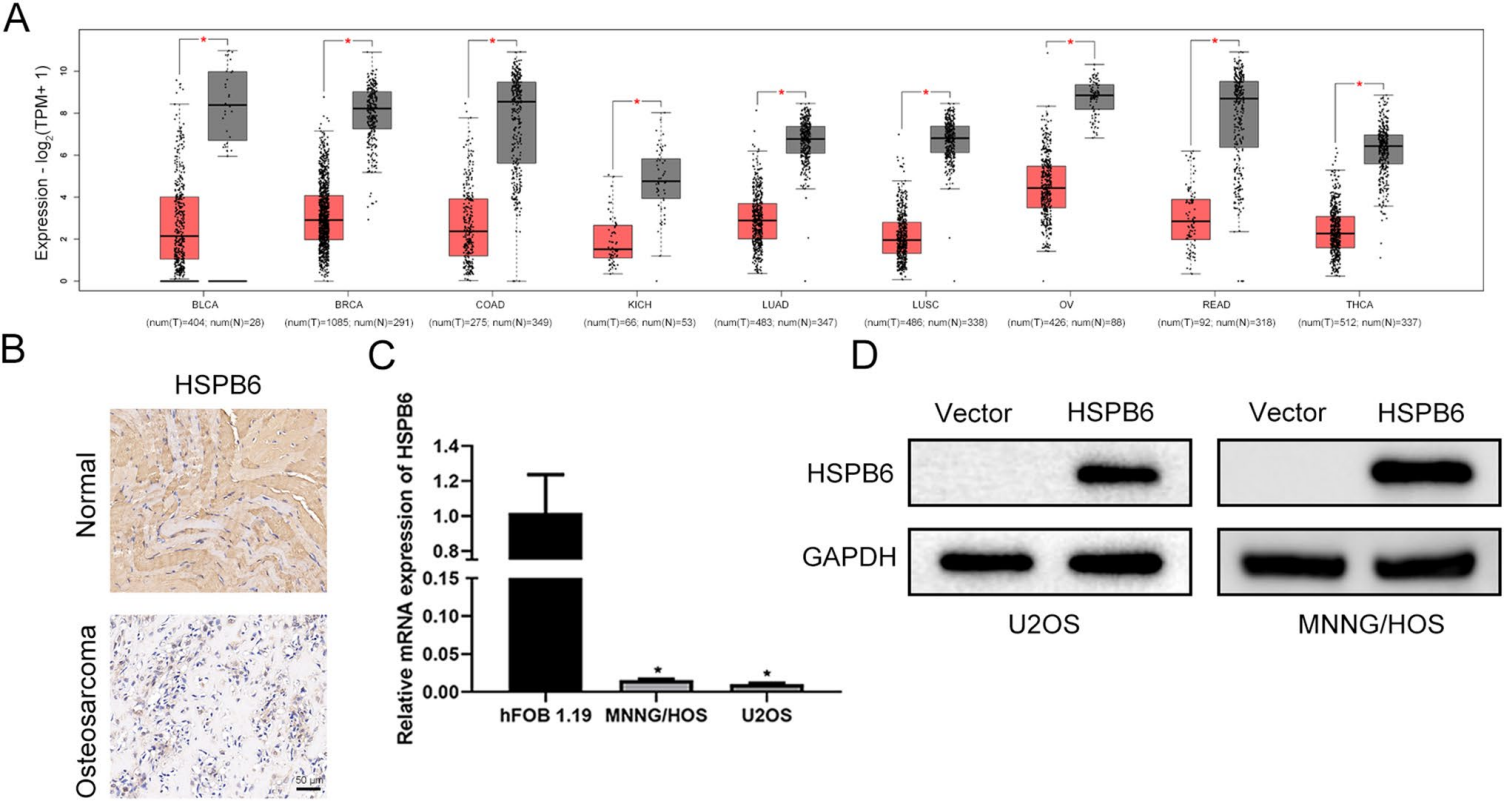
HSPB6 was down-regulated in several tumors and osteosarcoma. **A** The expression of HSPB6 in several tumors in the GEPIA2 database. **B** IHC investigate HSPB6 expression in osteosarcoma and normal tissues (*n* = 3). **C** RT-qPCR investigate the mRNA expression of HSPB6 in osteosarcoma cells and osteoblast cells.** D** Western blotting was performed to verify the overexpression efficiency of HSPB6. *P < 0.05

HSPB6 expression in different tumors was first investigated using Gene Expression Profiling Interactive Analysis 2 (GEPIA2) database [23]. It was discovered that the expression of HSPB6 is low in other tumors (Fig. 1A) And IHC assay show that HSPB6 expression decreased in osteosarcoma tissues (Fig. 1B). Next, RT-qPCR results suggested the significantly lower HSPB6 mRNA expression in osteosarcoma cells MNNG/HOS and U2OS compared with osteoblast cells hFOB 1.19 (Fig. 1C). Therefore, HSPB6 overexpression was constructed in U2OS and MNNG/HOS cells and validated with western blotting (Fig. 1D).

### HSPB6 overexpression suppressed proliferation and promoted apoptosis in osteosarcoma cells

**Fig. 2:**
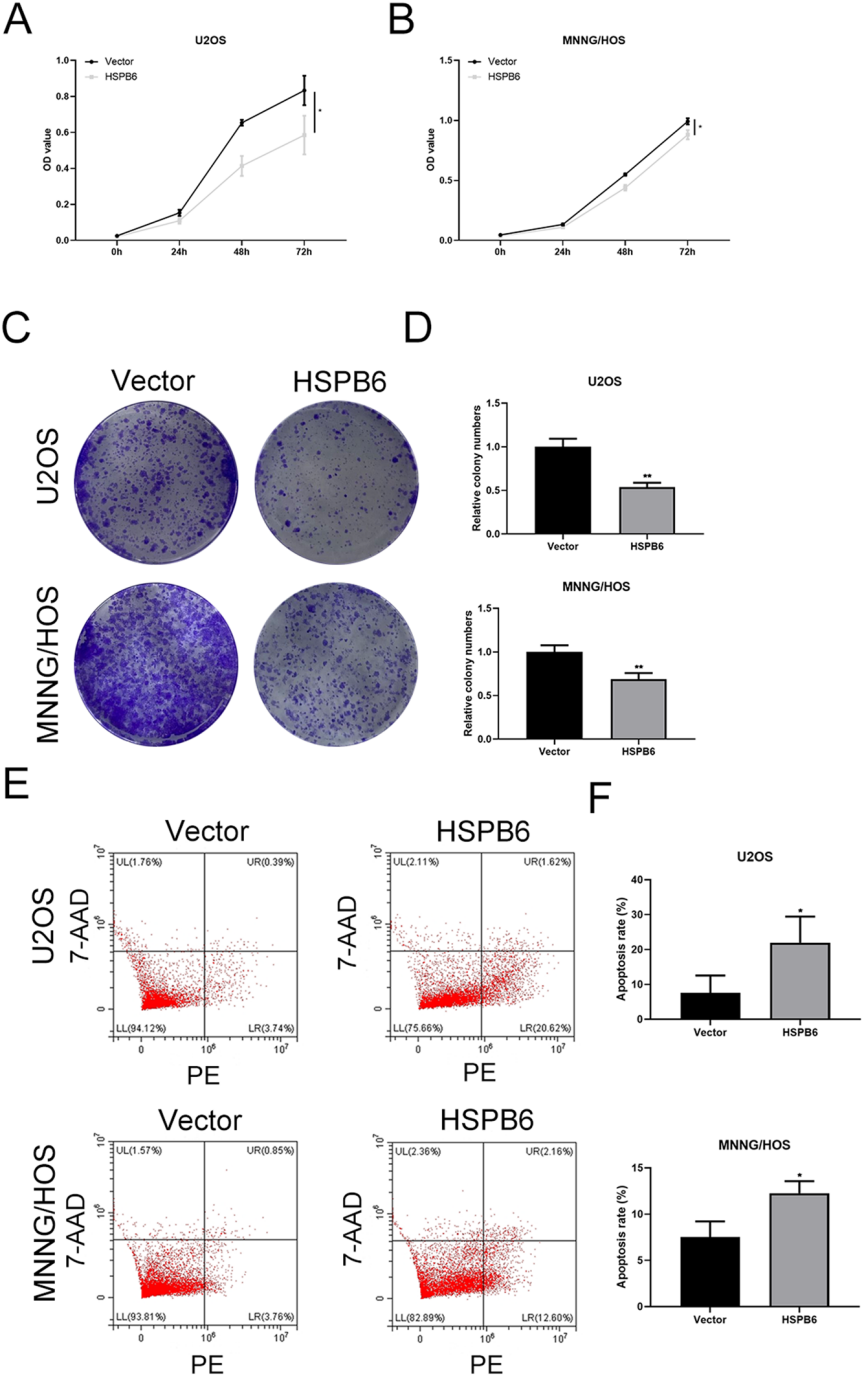
HSPB6 overexpression suppressed osteosarcoma cells proliferation and promoted apoptosis. **A**, **B** CCK-8 assay was performed to assess osteosarcoma cells proliferation. **C**, **D** Colony formation assay was performed to confirm the osteosarcoma cells proliferation ability. **E**, **F** Flow cytometry was performed for the detection of the apoptosis of osteosarcoma cells. *P < 0.05, **P < 0.01

The proliferation is regarded the most fundamental characteristic of cancer cells, and this equilibrium is broken in cancer cells [24]. CCK-8 assay suggested that HSPB6 overexpression suppressed U2OS and MNNG/HOS cells proliferation (Fig. 2A, B), and colony formation assay also indicated that the proliferation of U2OS and MNNG/HOS cells were reduced when HSPB6 was overexpressed (Fig. 2C, D). After the proliferation of osteosarcoma cells was determined, the apoptosis of U2OS and MNNG/HOS cells was quantified. As indicated by the result, HSPB6 overexpression significantly promoted apoptosis in U2OS and MNNG/HOS cells (Fig. 2E, F).

### HSPB6 overexpression suppressed osteosarcoma cells migration and invasion

**Fig. 3:**
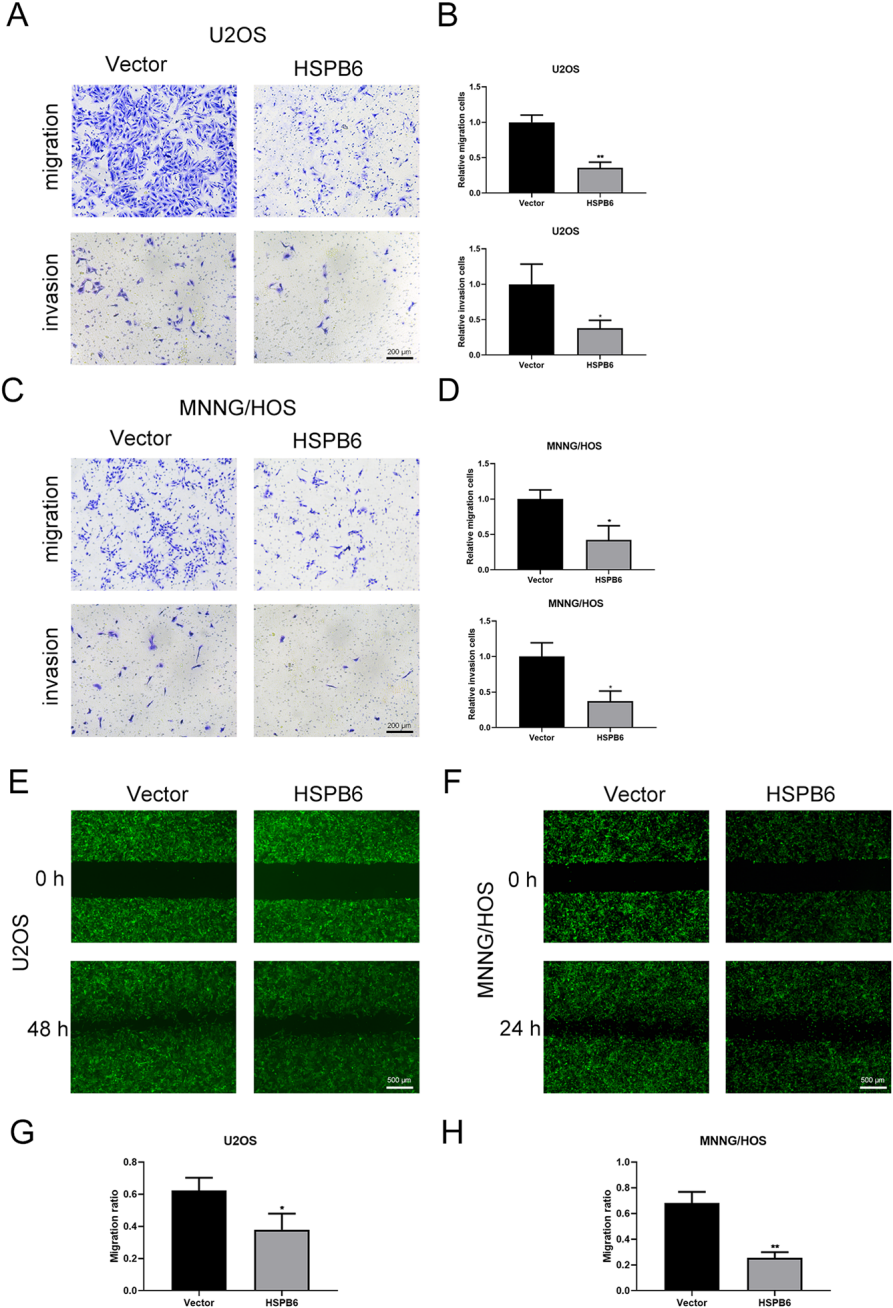
HSPB6 overexpression suppress osteosarcoma cells migration and invasion. **A**–**D** A transwell assay was used to confirm osteosarcoma cells migration and invasion. **E**–**H** A wound healing assay was performed to reveal osteosarcoma cells migration. *P < 0.05, **P < 0.01

Cell migration is a complicated and crucial step in the development of cancer [25]. Therefore, after the detection of osteosarcoma cells growth and apoptosis, we quantified the migration and invasion ability of osteosarcoma cells. The transwell experiment shown that HSPB6 overexpression dramatically decreased migration and invasion of U2OS and MNNG/HOS cells (Fig. 3A–D). Besides, wound healing results were consistent with the transwell results that HSPB6 overexpression suppressed U2OS and MNNG/HOS cells migration (Fig. 3E–H).

### HSPB6 overexpression suppressed the ERK signaling pathway

**Fig. 4:**
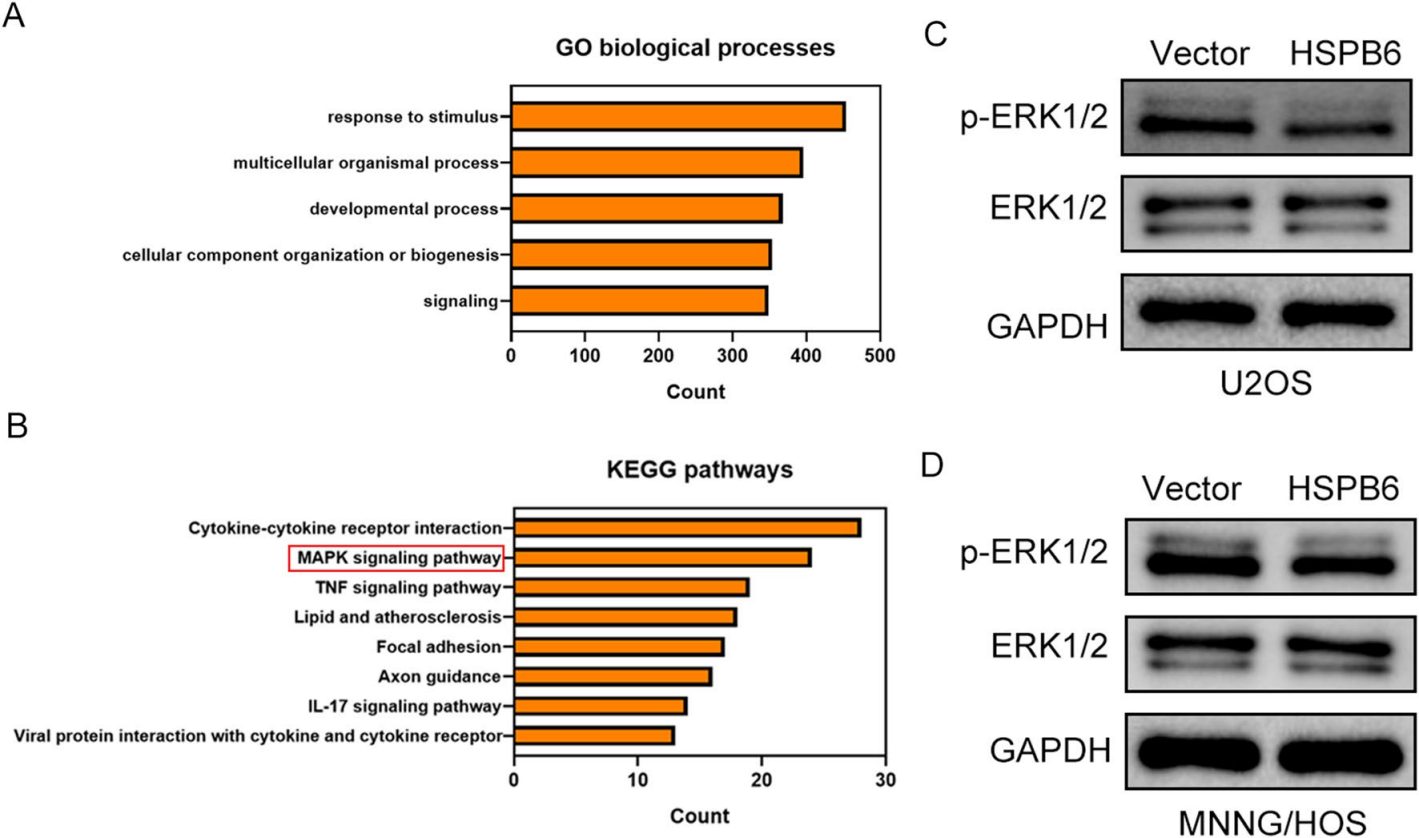
HSPB6 overexpression suppressed the ERK signaling pathway. **A** GO analysis of DEGs. **B** KEGG pathway analysis of DEGs. **C**,** D** Western blotting analysis for examining the protein expression of ERK in osteosarcoma cells

The RNA-seq was conducted after HSPB6 overexpression in osteosarcoma cells to search the potential mechanism and function of HSPB6 in osteosarcoma. As indicated by the result of the GO analysis, differentially expression genes (DEGs) played a certain role in several cellular processes (Fig. 4A). And result suggested the MAPK signaling pathway was enriched in Kyoto Encyclopedia of Genes and Genomes (KEGG) pathway (Fig. 4B). Furthermore, western blotting assay was performed, and result suggested that the protein level phosphorylation-ERK1/2 (p-ERK1/2) was suppressed in osteosarcoma cells when HSPB6 was overexpressed, whereas the protein level of ERK1/2 did not vary significantly (Fig. 4C, D).

### HSPB6 overexpression suppressed osteosarcoma growth and metastasis in vivo

**Fig. 5:**
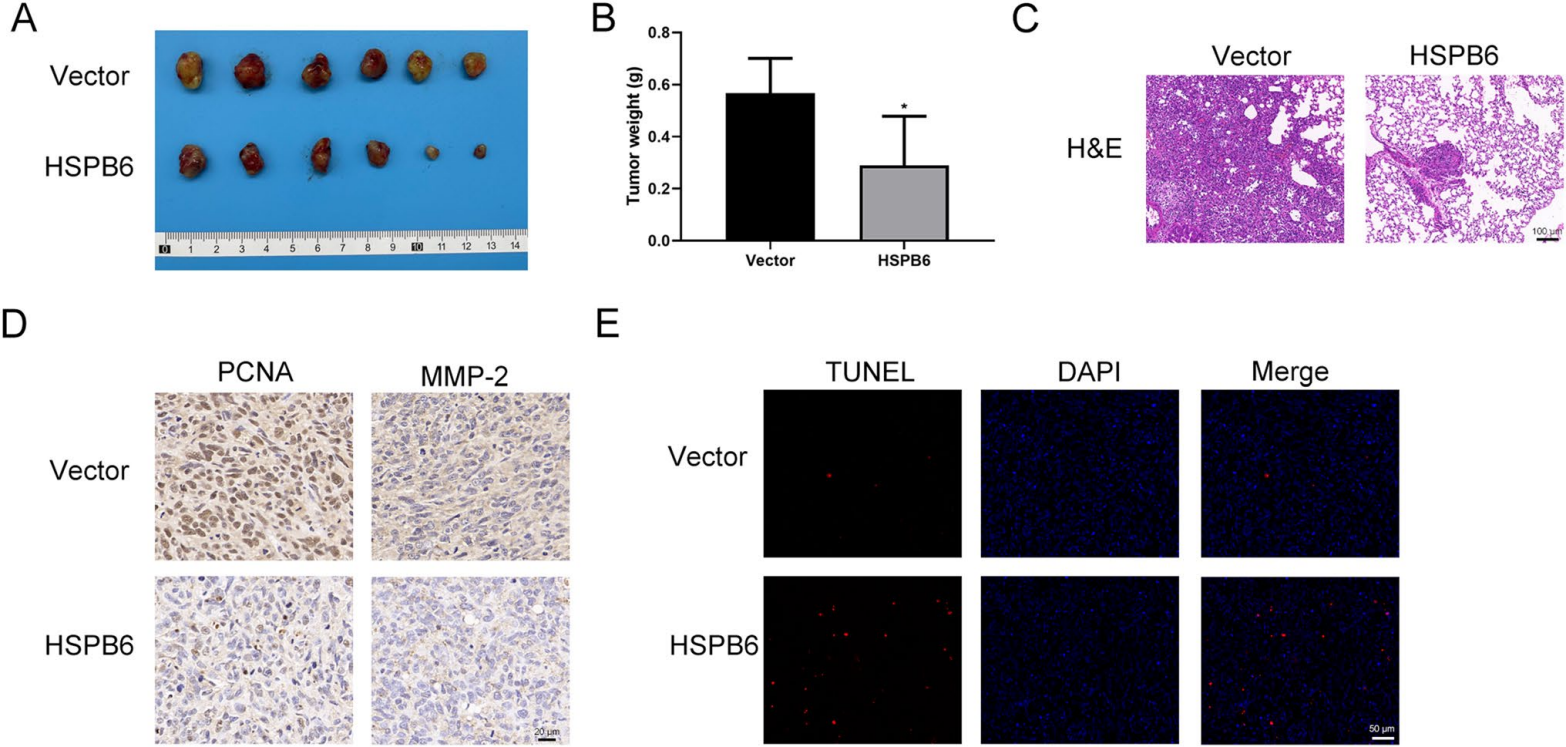
HSPB6 overexpression suppressed osteosarcoma growth and metastasis in vivo. **A** Tumor image were photographed. **B** Tumor weights were examined and presented. **C** H&E staining showed tumor lesions in lung tissue. **D** IHC analysis of PCNA and MMP-2 in tumors. **E** TNUEL staining was performed for the assessment of the apoptosis. *P < 0.05

The xenograft tumor model was employed to examine the effect of HSPB6 overexpression on the growth and metastasis of osteosarcoma in vivo. As revealed by the imaging of the osteosarcoma tumors, HSPB6 overexpression suppressed tumor development (Fig. 5A). In addition, the tumor weight was reduced (Fig. 5B). After tail vein injection in nude mice, overexpression of HSPB6 were reduced lung metastasis in vivo (Fig. 5C). Next, PCNA and MMP-2 expression was down-regulated following HSPB6 overexpression, suggesting the reduced ability of the tumor for migration and proliferation (Fig. 5D). TUNEL experiment also revealed that HSPB6 overexpression can promote tumor apoptosis (Fig. 5E).

## Discussion

Osteosarcoma is generally identified in long bones, comprising the proximal tibia and the distal femur [26]. The radiographic results are not pathognomonic for osteosarcoma, and a bone biopsy is required for the definite diagnosis [27]. Approximately 20% of osteosarcoma patients had identifiable metastases during diagnosis. 90% of metastatic sites are lung [28]. A considerable number of investigations on the cytogenetic and molecular components of osteosarcoma have achieved contradictory findings, such that their diagnostic and prognostic value remains restricted [29]. Despite the persistent attempt to ameliorate the diagnoses and prognoses of osteosarcoma patients, existing therapeutic interventions fall well short of satisfying the requirement for complete recovery, particularly for individuals with progressed phenotypes at the time of initial diagnosis [30]. On that basis, osteosarcoma diagnosis and treatment are subjected to challenges and have a long way to go, and other methods should be explored in the future for the treatment of osteosarcoma.

The heat shock response is orchestrated by heat shock proteins and heat shock proteins have been reported to play certain roles in cancer development [31, 32]. The heat shock protein 90 (HSP90) refers to an extremely conserved protein. Moreover, HSP90 expression is up-regulated in osteosarcoma. Subsequently, HSP90 knockdown can lead to the reduced osteosarcoma cells viability and migration and the dramatically reduced protein levels of p-AKT1, AKT1, Vimentin, as well as ki-67 [33]. Vimentin is frequently researched as a marker of cancer cell epithelial–mesenchymal transition (EMT) [34]. Moreover, heat shock proteins 70 (HSP70) take on critical significance in the formation of osteosarcoma. When MG63 cells are cultivated with cisplatin, HSP70 protein levels increased, HSP70 silencing can make MG63 cells more sensitive to cisplatin compared with the control group [35]. Except for HSP90 and HSP70, heat shock protein 27 (HSP27) has been identified in osteosarcoma formation. Besides, HSP27 can contribute to osteosarcoma cells resistance to Zoledronic acid (ZOL). As indicated by multiple studies, ZOL directly affects the proliferation and survival of osteosarcoma cells. While HSP27 is up-regulated in ZOL-resistant cell lines, suppressing HSP27 in resistant cells can lead to their improved sensitivity to ZOL. This resistance mechanism appears to be directly connected to the anti-apoptosis function of HSP27 in osteosarcoma cells, according to a study [36]. In comparison to HSP90, HSP70 and HSP27 in osteosarcoma, the effect of HSPB6 on osteosarcoma was unknown. The link between HSPB6 and osteosarcoma was thus examined using functional assays.

HSPB6 expression was down-regulated in several tumors by using GEPIA2 database. Next, the expression of HSPB6 was decreased in osteosarcoma tissues and HSPB6 mRNA expression was down-regulated in osteosarcoma cells MNNH/HOS and U2OS relative to osteoblast cells hFOB 1.19. Subsequently, HSPB6 was overexpressed in osteosarcoma cells, and its effect on biological behavior of osteosarcoma cells was observed. CCK-8 and colony formation results show that osteosarcoma cells growth was reduced following HSPB6 overexpression. The apoptosis can take on critical significance in tumor suppression, cell homeostasis, as well as vertebrate development [37]. Extrinsic and intrinsic routes have been reported as the two apoptotic processes. The extrinsic system functions via death receptors upon cell surface, whereas the intrinsic pathway, which is dependent on the mitochondria, is triggered by the deadly stimuli within the cell or the absence of growth factor signals [38]. Afterward, the apoptosis of osteosarcoma cells was identified through flow cytometry. After HSPB6 overexpression, osteosarcoma cells achieved enhanced apoptosis. Besides, transwell assay suggested that the migration and invasion of osteosarcoma cells was suppressed by HSPB6 overexpression, and wound healing experiment also found that HSPB6 overexpression inhibited osteosarcoma cells migration. As revealed by existing research, ERK signaling pathway played a certain role in apoptosis, proliferation, and migration of tumor cell [39]. Thus, the correlation between HSPB6 and ERK signaling pathway in osteosarcoma was explored. As indicated by the RNA-seq analysis, DEGs were primarily enriched in the MAPK pathway, and western blotting assay suggested that p-ERK1/2 protein expression in osteosarcoma cells was down-regulated after HSPB6 overexpression. Nevertheless, no significant variation was reported in the protein expression level of ERK1/2. The results suggested that HSPB6 overexpression can inhibit the proliferation, migration, invasion, and increased apoptosis in osteosarcoma by suppressing the ERK signaling pathway. After the in vitro investigations were completed, the in vivo effects of HSPB6 overexpression were assessed through animal experiments.

As indicated by the findings, HSPB6 overexpression inhibits osteosarcoma growth and lung metastasis in vivo. Existing research has revealed that besides DNA replication and repair, the proliferating cell nuclear antigen (PCNA) can also affect apoptosis [40]. Furthermore, PCNA has been reported as a promising diagnostic tool and prognostic indicator for evaluation of cancer therapy and disease progression [41]. Matrix metalloproteinases (MMPs) can be capable of degrading extracellular matrix (ECM) [42]. MMPs are governing a variety of cell biofunctions like cellular proliferative, differentiative, migratory, and apoptosis; MMPs activity is abnormally enhanced in numerous tumor cells [43]. Moreover, matrix metalloproteinase 2 (MMP-2) shows a correlation with tumors as well [44, 45]. Consequently, following the description of previous research, the expression of PCNA and MMP-2 were assessed through IHC assay, and HSPB6 overexpression suppressed the PCNA and MMP-2 expression. Lastly, TUNEL assay was performed to evaluate apoptosis, and apoptosis was improved after HSPB6 overexpression.

It is noteworthy that although we discovered HSPB6 plays the role in inhibiting the progress of osteosarcoma. There still exist some limitations in the present study. First, clinical data may help us to explore the relationship between HSPB6 expression and osteosarcoma patients prognosis. Second, if HSPB6 overexpression have an impact on osteosarcoma drug resistance, it may be better for clinical application. Finally, we expect to further explore mechanism of HSPB6 regulation of osteosarcoma in future studies.

## Conclusion

As indicated by the results of this study, HSPB6 was down-regulated in osteosarcoma, and HSPB6 overexpression was likely to reduce the proliferation, migration, invasion and lung metastasis of osteosarcoma and boost osteosarcoma apoptosis through the ERK signaling pathway. Based on previous research, HSPB6 may serve as a future therapeutic target for osteosarcoma.

## Data Availability

The datasets used during this investigation are accessible upon reasonable request from the corresponding author.
